# Bubble-Based Drug Delivery Systems: Next-Generation Diagnosis to Therapy

**DOI:** 10.3390/jfb14070373

**Published:** 2023-07-17

**Authors:** Mihaela Kancheva, Lauren Aronson, Tara Pattilachan, Francesco Sautto, Benjamin Daines, Donald Thommes, Angela Shar, Mehdi Razavi

**Affiliations:** 1 College of Medicine, University of Central Florida, Orlando, FL 32827, USA; mihaela.kancheva@knights.ucf.edu (M.K.); lauren.aronson@knights.ucf.edu (L.A.); francesco.sautto@knights.ucf.edu (F.S.); ben.daines@knights.ucf.edu (B.D.); dthommes@knights.ucf.edu (D.T.); 2 Biionix (Bionic Materials, Implants & Interfaces) Cluster, Department of Medicine, College of Medicine, University of Central Florida, Orlando, FL 32827, USA; taram@knights.ucf.edu (T.P.); angela.shar@knights.ucf.edu (A.S.); 3 Burnett School of Biomedical Sciences, College of Medicine, University of Central Florida, Orlando, FL 32827, USA; 4 Department of Materials Science and Engineering, University of Central Florida, Orlando, FL 32816, USA

**Keywords:** nanobubbles, microbubbles, drug delivery systems, gene delivery systems, imaging

## Abstract

Current radiologic and medication administration is systematic and has widespread side effects; however, the administration of microbubbles and nanobubbles (MNBs) has the possibility to provide therapeutic and diagnostic information without the same ramifications. Microbubbles (MBs), for instance, have been used for ultrasound (US) imaging due to their ability to remain in vessels when exposed to ultrasonic waves. On the other hand, nanobubbles (NBs) can be used for further therapeutic benefits, including chronic treatments for osteoporosis and cancer, gene delivery, and treatment for acute conditions, such as brain infections and urinary tract infections (UTIs). Clinical trials are also being conducted for different administrations and utilizations of MNBs. Overall, there are large horizons for the benefits of MNBs in radiology, general medicine, surgery, and many more medical applications. As such, this review aims to evaluate the most recent publications from 2016 to 2022 to report the current uses and innovations for MNBs.

## 1. Introduction

With increasingly innovative therapeutics being established each year, one such therapeutic gaining more attention is the use of nanocarriers and microbubbles (MBs). Although both are spherical shell-stabilized (e.g., lipid, polymeric, protein) structures consisting of a gas core, their corresponding sizes create different suitable applications [1,2]. For one, MBs are larger, with a size of about 0.5–10 μm in diameter, making them about the size of a red blood cell [3]. Because of the relatively large size of the particle, MBs tend to be unstable and usually require a shell that is made up of lipids, polymers, proteins, surfactants, or a combination of materials [3]. Each of these shells is of varying thickness. Furthermore, the gases with which MBs tend to be loaded are poor solvents, thus requiring a loading strategy to be utilized [3]. Ultimately, ultrasound (US) waves have been used in conjunction with MBs. They play a large role in the amplification of the biophysical properties of the US as contrast agents and molecular imaging for vascular targets. More specifically, they are known for increasing echogenicity, imaging sensitivity, and resolution when paired with the US. MBs are both therapeutic and diagnostic (“theranostic”) agents; thus, they can be used to deliver contrast for different types of diagnostic imaging, carry drugs, and deliver drugs in a more precise way than the millimeter scale dimension of ultrasound imaging [3]. However, their larger sizes compared to nanobubbles (NBs) hinder their ability to extravasate and penetrate past the cell membrane, causing them to accumulate within the perivascular space [4].

Similarly, NBs were also initially intended to be a delivery system of contrast agents [5]. Because they are on the nanoscale, though, NBs do not have the same limitations to the bloodstream that microparticles do. Within targeted drug delivery, NBs have strongly been shown to effectively disperse drugs and/or genes into tissues due to their small and compact nature [2,4,6]. Furthermore, research has delved into finding ways to utilize nanoparticles for targeted gene and drug therapy, especially with the improved stability of the bubbles as compared to the MBs [5]. Due to their small size, NBs are able to penetrate the pores within blood vessels and reach relatively difficult-to-reach places, such as tumors [5]. The ability of these particles to tend to gather in tumor tissues over normal tissue is called the enhanced permeability and retention (EPR) [7]. Similar to the MBs, NBs can also be used for imaging through the US contrast enhancement, but they offer the unique benefit of being able to leak out of tumor vasculature, thus allowing for visualization of the overall tissue and the possibility of surface marker targeting on tumor cells [8]. Thus, their longevity within the vascular system as a stable unit over time lends itself as a suitable target for the US-guided delivery [9,10,11].

Moreover, NBs have the unique ability to be able to “collapse” with the application of the US, causing the implosion of the bubble and allowing for the change in permeability of a cell membrane [7]. Exposure to the US is capable of facilitating drug/gene delivery through a heightened porosity of the cell membrane structure [2,4]. This occurs by “ultrasonic cavitation”, whereby the bubbles vibrate and grow from the acoustic energy of the US until they collapse [7]. The vigorous oscillations produced by higher acoustic pressures trigger the NB to expand, collapse, and release its contents. This creates a target site-specific release mechanism, enhancing the efficiency of the system in a process called inertial cavitation [4]. Specific US parameters can be optimized within the system to induce a target release reaction (e.g., intensity, frequency, time) [12]. The US cavitation also leads to the formation of gaps in the cell membrane of about 300 nm in diameter, allowing for local flow and shear stress on the nearby cells, thereby increasing the permeability of the vessel [7].

### Innovation in Micro/Nanobubbles for Drug/Gene Delivery and Imaging

Most recently, cancer research has been an expanding field of research, especially in regard to microbubbles and nanobubbles (MNBs). Although the overall rates of death associated with cancer continue to fall, the incidence of cancer is either leveling off in men or slightly increasing in women, showing that cancer-related treatments will continue to have a paramount effect in the battle against cancer [13]. The decades-long delivery of generalized chemoradiation and radiation has varying success for the different locations and types of cancer; however, such generalized therapy also comes with systemic and often debilitating side effects as non-cancer cells are also affected. As such, EPR can be utilized in order to administer passive targeted therapy to tumor tissues [7]. In recent years, NBs have been used for this role in EPR due to their small sizes but also because they can be altered in size to account for the pore sizes in the vessel [7]. For example, one study found that NBs made of folic acid (FA)-conjugated lipid and highly filled with artesunate (AL) (combine: FA-ALNBs) were effective not only in the destruction of adenocarcinoma cells in mice but also showed that bubbles did not have systemic effects around the cancerous cells [14]. More specifically, the researchers noticed that the cancer cells that were targeted with FA-ALNBs plus the US irradiation were able to uptake the most amount of the target drug in a dose-dependent relationship compared to their other groups that either did not use NBs, did not use the US, or did not use either [14]. Similarly, another study was able to administer curcumin, a drug used for prostate cancer that is usually administered orally with low bioavailability, through NBs to prostate cancer cells and, similarly, found cytotoxic effects specific to the prostate cancer cells [15]. Overall, a variety of new studies have used NBs for cancer treatments, including oxygen delivery, breast cancer treatment and imaging, doxorubicin delivery to anaplastic thyroid cancer, and paclitaxel delivery for lung cancer treatment [16,17,18,19].

Cancer, however, is not the limit to what MNBs are being researched as possible sources for treatment. In fact, MNBs are being looked at as a frontier for drug and gene delivery with imaging guidance. Gene delivery has been a focus of research for many years now. In 1984, the first viral vector was used to deliver vaccination to chimpanzees against hepatitis B [20]. Since then, gene delivery has continued to grow as a field, with MNBs at the forefront. Because of the large size of MBs, NBs tend to be utilized mostly for gene delivery as they are able to pass into tissues through the blood vessels [4]. Regardless, bubbles can be utilized for the delivery of nucleic acids, especially when combined with the US, in a variety of settings, including, but not limited to, cardiovascular (CVD) disease, central nervous system (CNS) disease, and tumors [4]. One application, for instance, uses gene delivery for osteoporosis-targeted treatment. It was found that once the silencing gene, Cathepsin K small interfering RNA (CTSK siRNA), was delivered with NBs, combined with osteoclast precursors, and activated with the US, there was suppression of osteoclastogenesis while also showing no significant cell death of the mesenchymal stem cells [12]. Other applications have utilized MNBs to maximize visualization and delivery. Gliomas, one of the most common brain tumors, tend to be difficult to treat because the blood–brain barrier (BBB) very effectively decreases permeability [21]. Thus, one study developed NBs loaded with Gambogic acid (GA)/poly(lactic-co-glycolic acid) (PLGA) conjugated with cationic lipid microbubbles (CMBs), which proved to be able to hold a high level of the targeting drug and contrast [21]. GA, a commonly used tumor chemotherapy, showed increased delivery by utilizing the US and the CMBs to open the BBB, thus allowing for the combined GA/PLGA to be delivered to the tumor and once again activated by the US [21]. Similarly, nanoparticle-shelled microbubbles (MMB-SiO2-tPA) were used, and the US was utilized to oscillate the MB to create a stepwise release of the nanoparticles filled with tPA, a drug that is used to break up clots but can result in dangerous systemic bleeds [22]. They were then able to use a magnet to target the MMB-SiO2-tPA to the site of the clot, which allowed for simultaneous imaging and delivery of the drug [22]. Other gene delivery applications of MNBs have included CD-TK double suicide gene therapy for bladder cancer, siRNA delivery to triple-negative breast cancer, and enhanced delivery of Fibroblast growth factor 21 for prophylaxis of diabetic cardiomyopathy [23,24,25].

The applications of bubbles in medicine have been an exciting sphere that is ever-expanding and growing. MNBs have essential components in the innovation of drug and gene delivery as well as imaging, but each bubble has its own characteristics and strengths. Thus, the applications of MNBs are ever growing and under rapid research over the past few decades. Because of this, this review will focus on the most recent (from 2016 to 2022) publications for innovation, along with giving a comprehensive background of MNBs.

## 2. Drug and Gene Delivery Overview

In the field of pharmaceutical and biomedical research, the development of new carrier systems for targeted therapy is revolutionizing the way medical diseases are treated [26,27]. Several formulations and devices, such as microspheres, hydrogels, MBs, and NBs, are being employed to deliver therapeutic agents for the treatment of a variety of diseases [28]. These new modalities are even surpassing conventional drug delivery methods both in accuracy and precision [28,29]. The effectiveness of these new carrier systems hinges on their ability to maintain adequate physical properties when exposed to the physiologic conditions of the body. They achieve this by bypassing certain biological barriers to sustain adequate therapeutic levels and can be designed to target-specific cell cycle phases or metabolic pathways [30]. Because of their small size, these carrier systems have also been shown to improve solubility and reduce toxic side effects.

Microspheres are polymeric particles with diameters ranging from 1 to 1000 µm, which are classified based on their composition of natural and synthetic materials [31]. They are comprised of a mixture of a drug dispersed in a polymer’s core, which is released via dissolution or degradation of the matrix. These particles have been proven to reduce dose frequency, improve bioavailability, and decrease overall toxicity [31]. The preparation methods of microspheres can also be modified to change the duration and the impact of the drug itself. However, poor reproducibility and variability in release rates of the same formulations have been noted. Despite their drawbacks, microspheres have been used in a multitude of drug delivery trials and therapies. Notable examples include encapsulated interferon-alpha for oral administration, cross-linked malonyl chitosan with encapsulated acyclovir for topical application, as well as possible delivery systems for vaccines [32].

Hydrogels are cross-linked networks of water-soluble polymers that can encapsulate medicinal cargo for drug delivery [33]. They are highly porous, which allows control of the cross-link density in the gel matrix. Hydrogels can also be altered into several different physical forms, such as nano-/micro-particles, films, and/or coatings [9]. Hydrogels offer such benefits as slow drug elution, biocompatibility with extracellular matrices, and easy diffusion across cell barriers. However, their hydrophilic core makes them incompatible with hydrophobic drug loading and causes unpredictable drug release rates, similar to microspheres [34]. This can pose a problem for drugs that necessitate extended releases, such as insulin or analgesics.

**Figure 1:**
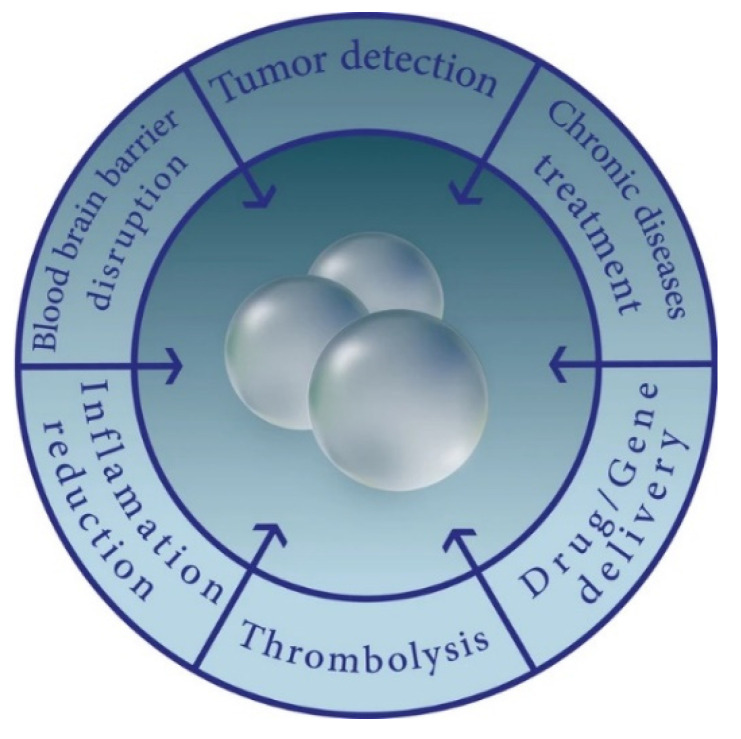
Bubble Applications. Visual representation of the different applications of nanobubbles. Reprinted with permission from Ref. [35].

**Figure 2:**
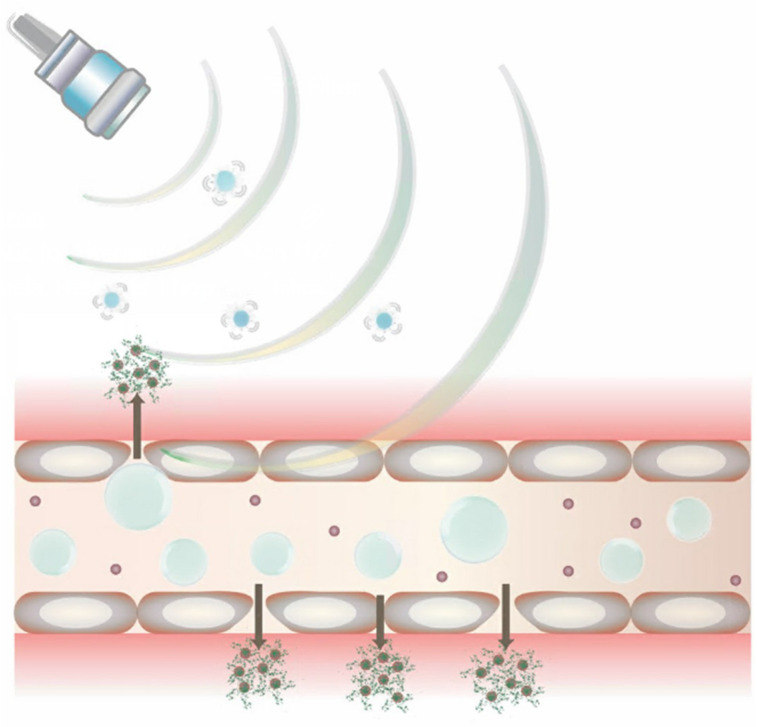
US bursting MNBs. The schematic shows the larger MBs being activated by the US and having their contents burst into the extracellular space. Similarly, the smaller NBs have escaped into the extracellular space where their contents may more directly interact once activated by US.

MNBs are multifunctional structures with novel properties and widespread application in medicine. These small gas-filled spheres consist of a gaseous core, a protective shell layer, and an aqueous/liquid external coat, allowing for both flexible and stable structures [35]. With respective diameters of 10–50 μm and <200 nm, they can infiltrate tissue walls with minimal interference from surrounding structures. In recent decades, they have gained increasing popularity as contrast agents for US imaging due to the ability to augment their behavior within the body via ultrasonic waves. This has proven to be useful for drug delivery, where both MNBs can be introduced to the body and modified to control their stability, absorption, release rate, and concentration at target locations [4,35]. MNBs are exceptionally capable as drug carriers with their high compressibility, low density, and their unique interactions with the US technology [36]. Given these advantages, along with their strong safety profile, speed, and low cost, US-directed MNBs have shown great promise in comparison with other drug delivery systems [4]. The use of MNBs in drug delivery is still in the preliminary stages, but further research is currently underway for its eventual approval to treat diseases in clinical settings. Ultimately, MNBs have a variety of applications possible (Figure 1) [35], especially through their activation with US (Figure 2). 

## 3. Bubbles in Practice

### 3.1. Bubble Types and Materials

**Figure 3:**
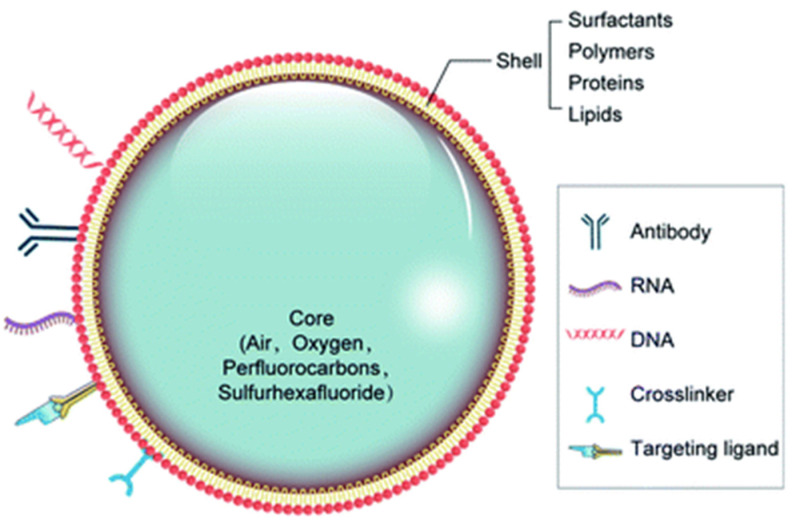
Nanobubble structure. Diagram of the many possible structures and compositions of a nanobubble. Reprinted with permission from Ref. [7].

NBs are traditionally considered to be a subset of the larger category of nanoparticles and nanocarriers, which often have similar base compositions and materials (though they vary vastly in function, density, and size) [37]. Most commonly, NBs are created as gas-filled liposomes containing phospholipids (i.e., phosphatidylglycerol or phosphatidylcholine) and gas (i.e., air or fluorocarbon) and often any other loaded particles or stabilizing compositions [38,39]. This might include materials, such as albumin, polymer, or lipid shells, to further stabilize the core and prevent early collapse, optimize its safety, and contribute to an efficient imaging [4]. Pluronics, for instance, are an assembled group of nonionic tri-block copolymers (which includes polypropylene oxide and polyethylene oxide) that interacts with the lipid shell and can reduce the size of MBs to NBs, improving echogenicity and stability [40]. Recent studies attempt to incorporate crosslinked polymers, which strive to improve the structural stability of the NB while reducing the outward diffusion of the gas core [40]. Other protein/polymer components of the shell may include cellulose, polyethylene glycol (PEG) composites, mesoporous silica, Poly (lactic-co-glycolic acid) (PLGA), lysozyme, avidin, and other polysaccharides, among others (Figure 3) [7]. The original pioneer MBs were Albunex, with a protein shell of sonicated 5% human serum albumin, and has been in clinical practice since 1993 as a myocardial contrast echocardiography agent [41]. The distinction of being gas-filled sets NBs apart from nanodroplets and nanoparticles, which consist of liquid and solid materials, respectively [38]. NBs contain a gas core, which is highly relevant to its echogenic properties and usage as a contrast agent for US and photoacoustic imaging [4]. Though both have applications in several fields (such as wastewater management, biomedical engineering, and medical imaging), MBs may face more limitations in biological systems, specifically in vascular and deep tissues, due to their challenges in penetrating the layers owing to their larger sizes [4]. However, there may be potential for the larger MB size to be useful in applications toward larger organs and regions in the body, as opposed to blood vessels. This might include well-perfused organs, such as the heart, kidney, and liver [42].

A uniform-size distribution, often labeled as monodisperse, is a desirable characteristic for NBs, especially in medical applications and settings, as it indicates more control and predictability in its usage. For instance, Helbert et al. found that monodisperse NBs have higher stability in vivo, increased imaging sensitivity, and a more uniform acoustic response in rat and pig studies [43]. The ideal preparation of uniform-size NBs remains undecided, though many novel methods in recent years have utilized a microfluidics approach or an extrusion approach in order to form monodisperse NBs at high concentrations [44,45,46]. Most commonly, however, MNBs can be prepared through sonication, a popular procedure due to its accessibility, in which they form due to the high-intensity US waves, leading to a cavitation process [41]. There are three existing types of NBs—surface, bulk, and micro-pancake NBs—which differ in where they are located and how they grow over time, though they share similar sizes [47]. Surface NBs are spherical caps on a solid–liquid interface and may have applications in the fabrication of nanostructures, nanopatterns, and nanodevices [48]. However, they are not stable long-term, while bulk NBs are spherical cavities submerged in liquid solutions (not on interfaces) that are intended to have long-term stability and survivability [49]. Finally, micro-pancakes are quasi-two-dimensional gas clusters resting upon a solid–liquid interface, which tend to exist in porous media systems [47].

### 3.2. Ultrasound-Mediated Drug and Gene Delivery Using Bubbles

NBs are directly relevant to medical applications due to their versatile role and usage in US imaging. The US imaging as a diagnostic imaging method offers some unique benefits, including non-invasive, painless imaging, cost-effective, and real-time images without the use of ionizing radiation, often harmful to the human body [50]. The US devices typically consist of a transmitter pulse generator, amplifiers, transducers, and accompanying digital systems and processors that display in real-time whatever is in contact with the transducer [51]. Typically, a water-based gel couples the US between this interface. Most commonly, its medical applications are diagnostic in nature and are used for gynecological, urological, cardiac, cerebrovascular, and abdominal examinations, among other regions [51]. More recently, the US has been shown to have therapeutic applications when used alongside NBs for drug delivery and as contrast agents. Though NBs are able to load drugs and serve as vehicles for delivery at target regions, they would normally remain in circulation and are unable to passively extravasate into deep tissues [52]. The US aids in this process by causing stable oscillations of the NBs by exposing them to acoustic pressure, a process known as cavitation [4]. When this process occurs at low acoustic pressure, it is known as stable cavitation, while when exposed to higher acoustic pressure, it becomes more unstable and is known as inertial cavitation [4]. This is the primary release and destruction mechanism that occurs and is useful for drug delivery and diffusion release to a specific region of interest. Thus, the longevity of the NBs can be controlled through cavitation as a potential parameter, in which bulk, long-lasting NBs can form through stable cavitation, while the latter induces more rapid destruction of NBs. Additionally, cavitation affects surrounding capillaries and cell membranes, causing them to become more permeable to the drugs released [50]. This process is known as sonoporation, referring to the formation of transient pores in local cellular membranes due to the MNB oscillation [53]. Of key importance is how NB stimulated and guided by the US improves upon the EPR effect. The EPR effect is a mechanism and phenomenon in which protein compounds and conjugated drugs are able to accumulate into highly vascularized tissues and effectively target the region of interest, as often observed in cases of inflammation or cancer [54].

US cavitation leads to acoustic streaming, a mechanical phenomenon in which the oscillating flow leads to non-linear fluid flow that can displace small molecules and ions in its path [55]. Together, these two phenomena form the basis for US-caused thermal effects, which typically increase with higher US intensity. This heating occurs from the dissipation of the mechanical energy transforming into thermal energy and may have applications in the thermal ablation of target tissue and tumors [56]. Another potential effect of the collapse of MNBs involves the formation of free radicals due to an elevated chemical potential around the gas–water interface [57]. Ultimately, when using the US in conjunction with NBs, therapy and diagnosis can be personalized by understanding the specific mechanism of the desired US action, for instance, modifying to a lower intensity and frequency for sensitive regions and soft tissues.

A wide range of drugs can be delivered through NBs, which include small interfering RNA (siRNA), micro-RNA, antisense oligonucleotides, plasmid DNA, anti-cancer agents, and proteins, among others. Additionally, there are typically three classifications of regions seen in the NB drug delivery studies—tumor sites, the open blood–brain barrier, or vascular sites [36]. NBs are loaded with drugs in two primary ways: by associating the drug with the shell; or by encapsulating a hydrophobic drug inside the gas-filled core [58]. Additionally, NBs can target specific ligands by also displaying antibodies on their surfaces. The mechanism of drug delivery using NBs is an amalgamation of steps following its US stimulation. Typically, MNBs are injected intravenously, entering the blood circulation [59]. The placebo group in several studies, in opposition to the experimental group, tends to be a saline solution injection. However, without guidance from the US, the MNBs are without direction—with the usage of a focus US, the permeability of a particular region of interest can be increased through the EPR effect, allowing access to the drug-loaded bubbles to then release through stable cavitation and sonoporation [59]. Finally, the lipid-shelled MNBs are biodegradable, following release and destruction as it is taken up into local cells through endocytosis, lessening the total cytotoxicity as a procedure [58]. While this process is occurring, if the subject is imaged using the US, the NBs would be visible due to their gas-filled core expanding over time, leading to higher compressibility than soft tissue and reflecting the US better as a result [60].

The total effectiveness of the mechanism must involve the consideration of specific parameters, such as the composition, size, and polydispersity of the NBs in synthesis, the intended drug loaded into the NB, the selected US intensity, and the region of interest, among others. Low-intensity US tends to be less toxic in subjects, while high-intensity US could rupture capillary vessels, displacing the MNBs into the unwanted tissue regions [61]. Thus, often, this warrants trial-and-error studies that guide future optimization steps. For example, Prabhakar et al. coated their sample MBs with drug-loaded nanocarriers that formed pendant-shaped complexes in an attempt to improve therapeutic payload but faced a cost of reduced therapeutic efficacy as the complex size limited their extravasation to the tumor region [60]. As the field of nanoparticle-based drug delivery is still quite novel, many research studies attempt to formulate a novel approach to treatment, improving upon predecessor studies and their parameters. For instance, Xie et al. introduced a new strategy for improving targeting and delivery efficiency to tumor sites by loading cell-penetrating peptides (CPP)-camptothecin conjugating into their NBs, which later showed effective cellular uptake in HeLa cells in vitro and in xenografted mice when compared to a normal CPT injection group [62]. CPPs are cationic peptides that are able to interact with and facilitate the uptake of therapeutic agents by cells via interactions with the negatively charged plasma membrane. The main drawback of CPPs, however, is that these peptides are non-specific and, thus, would not be specific to a particular region and cells of interest. When combined with the US-mediated targeted drug delivery, however, the lipid-shelled NBs successfully masked CPPs’ non-specific cationic interactions and were able to release the CPPs at their specific regions of interest. The composition of the NB in this study was adapted for US stimulation, containing the US-sensitive agent perfluorobutane [62]. Another approach by Song et al. used magnetic properties to guide NBs, which also improved cytotoxic effects in addition to forming multimodal applications within US, MRI, and photoacoustic (PA) trimodal diagnostic imaging [17]. These NBs were decorated with Herceptin and contained ultrasmall superparamagnetic iron oxide (USPIO) and paclitaxel (PTX) (called PTX-USPIO-HER-NBs); the bubbles were synthesized through a modified double-emulsion evaporation process combined with the carbodiimide technique for peptide bond formation. USPIOs offer several advantageous properties, such as low toxicity, small particle size, and high magnetization, which have been used as MRI contrast agents and can produce PA signals following laser energy absorption and its subsequent thermal expansion [17]. Furthermore, a perfluoropropane gas core was used in the NBs due to their US contrast agent potential; the NBs were PLGA-based, as the PEG chains were resistant to external factors, such as heat, enzymes, and changes in pH, which allowed them to maintain the biological activity of drugs and extend their half-life in the blood [17]. Though there remain challenges and improvements to be made with loading single drugs into MNBs, there are potential benefits to exploring multi-drug delivery mechanisms using NBs, especially when considering agonistic and antagonistic relationships of drugs. In osteoporosis treatment, for instance, the bisphosphonate alendronate could be used as a bone resorption inhibitor and a bone-targeting molecule; if conjugated with Cathepsin K-targeting siRNA to disrupt resorption, it may increase the overall effectivity of osteoporosis treatment [63]. In conjunction with these targeting molecules and their selective properties, a perfluorocarbon gas core NB can be used in its synthesis due to its US-responsive nature and contrast agent utility [12]. This concept of loading multiple drugs in combination therapy and delivery was explored by Şanlıer et al., who presented a novel mechanism for dual-drug delivery of pazopanib and pemetrexed for non-small cell lung cancer treatment by conjugating both of the drugs to a designed hexapeptide, which was then bound to magnetic nanoparticles within the liposomes [64]. Very few to none of the MNBs studies surveyed have explored multi-drug delivery systems, which is logical considering the challenges and several studies surrounding singular drug-loading applications. However, therapeutic and diagnostic multi-drug NB delivery systems are certainly an application with high potential if applied wisely to the most consequential diseases and health issues. 

### 3.3. Bubbles in Image-Guided Drug Delivery

Drug delivery has two main aims, which are to deliver the drug to the target tissues that you want and minimize toxicity to surrounding tissues where you do not want the drug to be, therefore, maximizing benefits and reducing side effects. One growing field of research focused on drug delivery is the image-guided delivery of drugs with the help of MNBs. Imaging, when related to bubble drug delivery, most frequently comes in the form of the US. The US is an effective option for several reasons. It limits exposure to radiation, is cost-effective, easy to use, and provides real-time imaging [4,65]. US-mediated drug delivery is not completely understood, but according to an article by Wei, there are two main mechanisms, thermal and non-thermal effects [66]. According to Wei et al., the thermal effect is due to the vibration or thermal energy that the US provides. It increases the thermal energy of cells within the treated tissue. This increase in thermal energy causes an increase in cell membrane and blood vessel permeability and access to tissues that otherwise may be difficult to treat, such as tumors. The non-thermal effects are mainly associated with cavitation and subsequent drug release—a phenomenon seen in both MNBs. Cavitation can be further subdivided into inertial and non-inertial cavitation. Inertial cavitation refers to a sustainable cycle of stretch and relaxation on the bubbles, which is enhanced by the addition of the US. Inertial cavitation is when bubbles suddenly collapse, creating micro streams and free radicals. The drug delivery, as caused by the addition of the US, is thought to be due primarily to shear stress and shockwaves from the bubble collapse. Cavitation has secondary effects of increased vascular permeability and sonoporation, which increases openings in US-exposed cell membranes leading to an enhanced drug uptake [66].

Once the drugs have been loaded onto MNBs, they must be delivered to desired tissues. There are two main forms of drug delivery using MNBs. Those are systemic and targeted. Systemic delivery is mainly for gene delivery; however, this section will focus on targeted drug delivery, which frequently is achieved by focused US. One older technique for targeted delivery is to use ligands [65]. These ligands can include antibodies, carbohydrates, and peptides, but they must be intravascular since the bubbles stay in the intravascular space until they release their contents. Ideally, these ligand markers would only be expressed in the pathologic areas of the body to increase specificity [65]. More recently, antibody-modified bubbles have been developed that are able to target certain diseases or disease processes [4]. For example, past targeted ligands include vascular adhesion molecule-1 for Crohn’s disease, matrix metalloproteinase-2 or intercellular adhesion molecule-1 following myocardial infarctions, and even MAP-2 antibodies to prevent apoptosis following a spinal cord injury [67,68,69].

Other than ligand-guided delivery, bubbles can be directed by focused US in conjunction with lipophilic MBs that more easily cross the BBB [70]. The BBB has historically been a difficult membrane to cross and only allows molecules that are small and lipophilic [71]. Although MBs and US are able to temporarily disrupt tight junctions in brain endothelium, they allow for greater permeability of drug-loaded MBs. While this technology is promising, it is still under investigation for clinical applications, and more optimized bubbles need to be designed to better penetrate the BBB [68,72]. MBs have also shown promise in future cancer treatments [73]. Specifically, showing SonoVue^®^, which employs MBs with the US that enhanced the number of gemcitabine treatments pancreatic patients could undergo. There have also been studies showing that MBs and US could increase the sensitivity of gastric cancers to chemotherapy [74].

### 3.4. Challenges in Bubble-Based Image-Guided Drug Delivery

While there are several promising applications of MBs and the US as a tool for drug delivery, there are problems that need to be solved first. Currently, MB size is difficult to control [66]. Their size directly affects their echogenicity, which is their ability to compress and oscillate upon exposure to the US frequency [73]. This, in turn, changes the MBs’ response to the US and can make using them unpredictable. There is a number of techniques that are being developed to standardize MB size and, therefore, delivery, but right now, their high cost and low yield make it difficult to apply widely. New bubbles may also need to be developed in order to deliver drugs better since larger bubbles and better imaging capabilities are more stable and can hold more drug products but for the tradeoff of making them more susceptible to the body’s own immune system. NBs also look promising for US-directed drug delivery due to their small size, which increases intracellular uptake compared to MBs. However, currently, NBs are fragile and subject to destruction by shear stress and rapid gas diffusion. This fragility makes it difficult for NBs to be separated from free drug solutions without reducing the bubble yield. So, while NBs can be used for drug delivery, more stable formulations could help reduce the amount of free drug injected and, therefore, reduce off-targe effects [75]. Advancements need to be made to increase NB stability before they can be used as a drug delivery device [66]. Finally, because MNBs, as vessels of drug delivery, are so new, we do not have sufficient information on their long-term health effects.

### 3.5. Bubbles in Gene Delivery

Gene delivery has come under public focus since the advent of the mRNA vaccine for the COVID-19 pandemic, which utilized lipid nanoparticles to enter the cells and release mRNA that would be translated into the spike protein of the virus, which could then elicit the cellular immune response to form antibodies [76]. As described in previous sections, NBs combined with the US can have a role in focalizing the release of nucleic acids to specific parts of the body for therapeutic and diagnostic reasons. In this section, we will describe different methods of gene delivery and how they compare with bubble technology.

The current literature on gene delivery methods separates them into two categories, viral and non-viral. Other reviews have described in detail the various types of viral vectors utilized along with the advantages and disadvantages [77,78]. To summarize, viral vectors include adeno-associated viruses, lentiviruses, and adenoviruses, with the most commonly used for in vivo delivery being adeno-associated viruses. The adenovirus vector was recently used in the Johnson&Johnson vaccination for COVID-19 for delivery of spike proteins into the cells [76]. Advantages of viral vectors can include higher rates of transfection, promotion of long-term expression, and protection of cargo from degradation, though disadvantages include higher rates of immunogenicity in the population with adeno-associated viruses and adenoviruses, inability to have short-term effects, and high risk of off-target effects due to infective mutagenesis [77]. Certain serotypes of viral vectors can be utilized to target certain parts of the body, such as MyoAAV, having a significantly higher affinity for muscle cells and can be used at lower doses [78]. Given the risk for mutagenesis with viral vectors, research is transitioning into methods of directing gene delivery through non-viral means, of which bubble technology will be discussed.

Descriptions of MNB delivery systems have been given in previous sections. With relation to gene delivery, bubbles that are negatively or neutrally charged have low affinity for negatively charged nucleic acids, such as DNA or RNA, so cationic MNBs have been developed with greater loading capacity due to the ability of electrostatic interactions to take place with gentle mixing [1,4,69]. Given the NBs’ higher retention time and ability to diffuse across the vasculature, unlike larger MBs, it would seem more advantageous to utilize NBs to target deeper tissue layers of the body [79]. However, NBs may have reduced control over targeted delivery once beyond the epithelium and result in an unequal distribution [79]. Yet, one key aspect of certain NBs is a phospholipid coating, which, when added with a PEG layer, allows for the coupling of bubbles with antibodies that assist in targeting locations in the body while also decreasing any excess positive charges to prevent degradation [1,79,80,81]. Other researchers have begun utilizing either nucleic acid-based or albumin-based coating [4,82]. Given the previous success with viral delivery systems, there have also been attempts at combining viral vectors and MBs with a biotin–avidin bridge method [83]. This section will, thus, evaluate different studies utilizing US-mediated MNBs delivery to further illustrate how they can be used in gene delivery.

**Figure 4:**
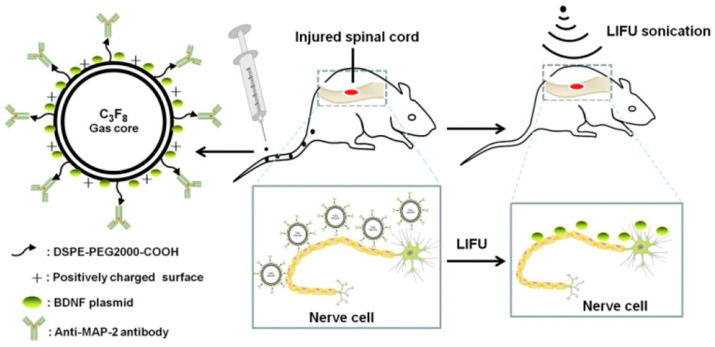
The structure of mAbMAP-2/BDNF/CNBs and uses. The illustration shows the structure and function of mAbMAP-2/BDNF/CNBs in targeted gene therapy for nerve cell repair. Reprinted with permission from Ref. [69].

Takahashi and Negishi, in their review, cited many different examples of gene delivery via US-mediated MBs, including delivery of Ang-2 plasmid to the brain, utilizing peptides as guides to increase perforation in ischemic tissue [1]. A more recent example of a similar finding was given in 2018 with MAP-2 used as the neuron-specific guide for an antibody and BDNF, which has been shown to promote growth and synaptogenesis in neurons, utilized as the gene to be delivered to promote neuronal regeneration after spinal cord injury [69]. The NBs used had a perfluoropropane core and a PEG lipid shell linked with an anti-MAP-2 antibody and mixed well with a green fluorescent protein (GFP) labeled BDNF plasmids (Figure 4). The in vitro portion assessed effects of NB complexes compared to control under the US irradiation of 1.5 W/cm^2^ on spinal neurons, which found higher degrees of fluorescence, BDNF mRNA expression, and decreased neuronal apoptosis in the full complex group. The in vivo portion assessed the complexes within mice that had a standardized contusion injury at the 10th thoracic segment, with drug injection every 12 h and US irradiation of 4 W/cm^2^ for 5 min over 3 days. The results successfully indicated that the mice that underwent full complex treatment had normal morphology, more regeneration, less necrosis, and more Nissl bodies compared to the control, with significantly higher BDNF mRNA expression. One caveat with this research was the assertion that there were reports which showed that low-intensity, high-frequency US might increase levels of BDNF, GDNF, and VEGF to treat cerebral damage, so future experimentation would need to address the effects of US on neuronal repair. It did, however, confirm that the technique is a promising way of delivering genetic interventions to treat disease.

**Figure 5:**
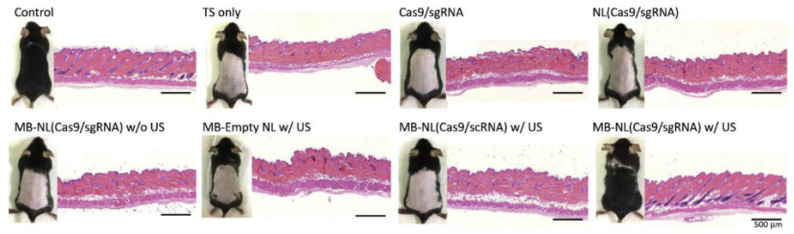
Hematoxylin and eosin (H&E) staining of mouse skins following treatment for 7 weeks. Treatment with US-activated MB-NL(Cas9/sgRNA) did not show a difference in density of the hair follicles when compared to controls, but the other treated mice group did show differences in density. Reprinted with permission from Ref. [85].

**Figure 6:**
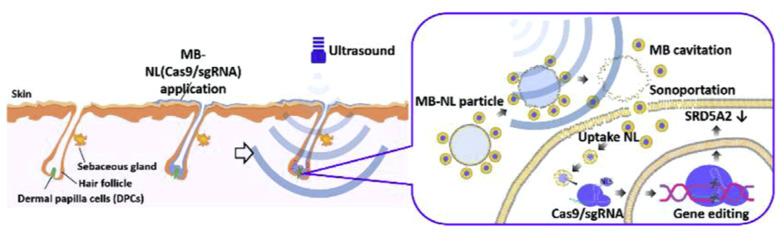
Schematic of US exposure to nanobubbles. Following exposure to US, the MB-linked particles released their solution, allowing the particles to penetrate the dermal papilla cells (DPCs). Reprinted with permission from Ref. [85].

One significant advancement in genetic interventions is the rise of Clustered Regularly interspersed structured repeats (CRISPR)/Crispr-associated protein (Cas) 9 technologies utilized for genome editing therapy, though the efficacy of direct interventions has been low; thus, multiple carriers have been developed to assist in the delivery of the editing complexes, including viral vectors, gold nanoparticles, and the discussion of today US-mediated MB technology [78]. A study in 2019 utilized a cationic MB combined with low-frequency US of 1 MHz at 1 W/cm^2^ to transfect human endometrial cell lines (HEC-1A) in vitro with a CRISPR/Cas9 plasmid specifically to knock out Epidermal growth factor 2 (C-erbB-2), which is found to be overexpressed in endometrial cancers, and they found that there was significantly lower expression of C-erbB-2 with US-mediated MB transfection [84]. The results of that study, however, could not indicate whether the US-mediated technique was the cause of the increased transfection or whether the specific single guide RNA (sgRNA) used contributed to reduced expression. However, another study was able to assess both the in vitro and in vivo effects of US and MB-mediated CRISPR/Cas9 plasmid transfection to knock out steroid type II 5-alpha reductase (SRDA52) to combat alopecia [85]. The findings for the in vitro portion were similar to the previously discussed article in that the US-mediated MB treatment showed significantly reduced expression relative to the control. For the in vivo portion, the study was able to illustrate that delivery of direct CRISPR/Cas9 plasmids was insufficient to penetrate cellular complexes to regenerate hair and that only the combination of US application and MB complexes were able to show initiation of hair growth in depilated mice (Figure 5). This was important considering that MB delivery with CRISPR/Cas9 alone was still inefficient in causing the results and that the US application allowed for the sonoporation of those MBs into dermal cells (Figure 6). Unfortunately, the study was not able to assess the long-term adverse effects of the treatment in the mice tested, and while the off-target effects observed were less than 3%, further experimentation would need to be performed with repetition and long-term observation prior to conclusions being made in favor or against MB delivery with US mediation of CRISPR/Cas9.

**Figure 7:**
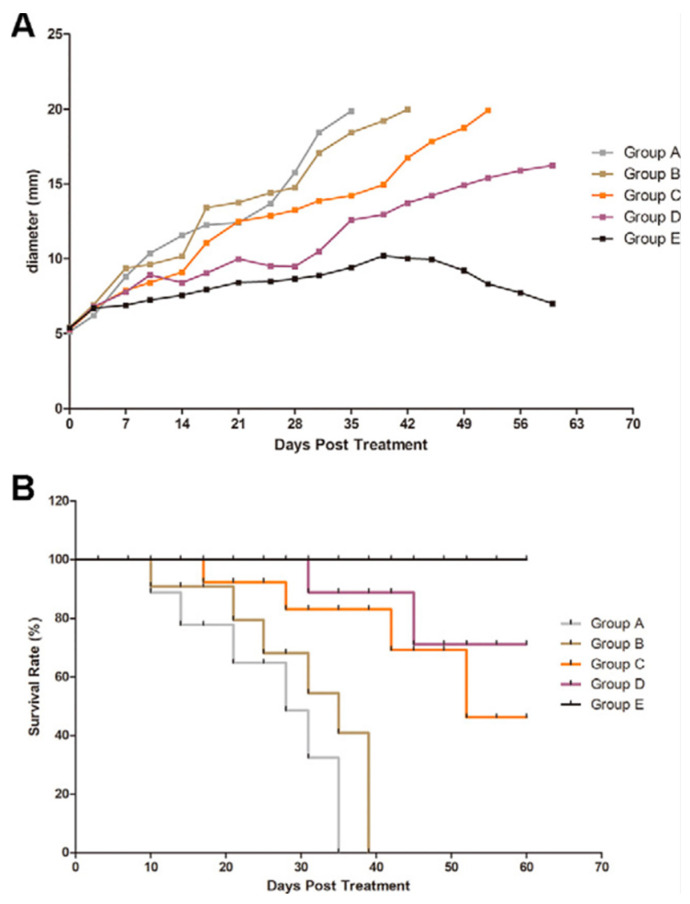
Survival and Tumor Growth in Mice. In both figures, the groups represent the treatment of 0.5 cm tumors in mice. Group A, blank; Group B, negative control siRNA duplex and Lipofectamine 3000; Group C, NET-1 siRNA duplex and Lipofectamine 3000; Group D, NBs and NET-1 siRNA duplex; and Group E, NET-1 siRNA-conjugated novel-targeting NB. (**A**) Depicts the effects of the different treatment groups A through E were exposed to on the sizes of the tumors. (**B**) Depicts the effects of the different treatment groups A through E on survival rates. Reprinted with permission from Ref. [86].

As noted in the former study from the previous paragraph, MBs as gene delivery mechanisms have been largely studied in the treatment of different cancers, given that overexpression or underexpression of certain proteins may drive carcinogenesis. To illustrate that concept, we will be utilizing research into the treatment of hepatocellular carcinoma (HCC) through the delivery of nucleic acids via US-mediated MBs. This study presented targeted delivery of small interfering ribonucleic acids (siRNA) to suppress the expression of NET-1, found to be overexpressed in HCC cells, with an antibody to Glypican-3 (GPC3), that is expressed in HCC but not normal hepatocytes [86]. They compared different delivery mechanisms, including biotin–avidin-created NBs conjugated with a NET-1 siRNA complex (Figure 7, Group E), which ultimately showed the most significant decrease in tumor growth within mice that were subcutaneously injected with HCC tumor cells, with all groups being irradiated with low-frequency US of 1 MHz of 1 W/cm^2^ (Figure 7). Further, Group E showed a 100% survival rate, whereas all the other groups showed some or a complete decrease in survival during the study (Figure 7B). While NBs allow for greater penetrance into deeper tissue layers and vasculature, this experiment only assessed the effects of the treatment on subcutaneous tumors. A targeted approach to HCC in situ may warrant further experimentation, given reduced control and unequal distribution [79,86]. Future experimentation may venture into the work of phase shift nanodroplets, which have recently shown promising findings in microRNA (miRNA) delivery in treating the HCC [81,87]. These nanodroplets differ from NBs in that they have the advantage of being in a superheated state and have longer circulation times in vivo [81].

Overall, MB technology has been a promising method for gene delivery that is gaining traction as the cons of viral vector technology become apparent, with MBs having more protection from the immunogenic response, less mutagenesis risk (aside from those that may present with CRISPR/Cas9 interventions), and when mediated by US irradiation, can be targeted to specific tissues in the body [79]. One key difference between MB technology and NB technology in this regard is that the deeper tissue layers can be penetrated with the latter, though with more complex vascularity, there exists a continued problem of unequal distribution [79]. While the studies described above have shown success, there is an inconsistency in how the bubbles are constructed and the method of US irradiation used. Su et al. mentioned that high ultrasonic energy needed to induce cavitation of target cells for NBs to function might inadvertently damage tissue, and the rapid release of those genes means that there is no continuous release (though this may be seen as an advantage in certain conditions) [7]. Certain studies used a low-frequency US of 1 MHz at 1 W/cm^2^, while others utilized a greater intensity at 4 W/cm^2^, compounded by inconsistency in how far away the probe must be from the targeted tissue or how long the treatment must be; thus, future research should have a focus on optimizing the US techniques for the MB-mediated gene delivery [7,78,86,87]. Otherwise, more research will be needed with long-term observation of in vivo interventions to assess for off-target effects of gene delivery. 

## 4. General Bubble Administration

MNBs have several different potential functions in the body, as discussed earlier. In order to achieve these effects, the bubbles can be administered in different ways to target different areas of the body. The main mechanisms of delivery include systemic, oral, and loco-regional.

The most simple version of MB delivery is through injection directly into the bloodstream [88]. This is often administered in small amounts, less than 1 mg per injection, and is frequently used to enhance the US imaging in a patient. The bubbles last in circulation for only a few minutes and have been shown to have a good safety profile [88]. Microbubbles can also be injected systemically for drug delivery and, using the US, can be targeted to specific tissues [89]. This can be administered in either a single bolus injection or a constant infusion. 

Oral administration of microbubbles has interesting possibilities for the delivery of drugs, immunotherapies, and more. MBs can be administered with an electrospray that allows MBs to adhere to the stomach wall [90]. Prolonged adhesion to the stomach from the MBs allows for better absorption of drug products. Additionally, MBs can be loaded with antigens and delivered orally [91]. Oral delivery of antigens helps increase the immunogenic response of mucosal tissue with which they come in contact compared to parenteral vaccination. MBs could provide an avenue for antigens to survive the harsh environment of the GI tract while still providing immunological protection. Similar MBs-associated antigen administration strategies have shown effectiveness in the intranasal introduction of allergens [92].

**Figure 8:**
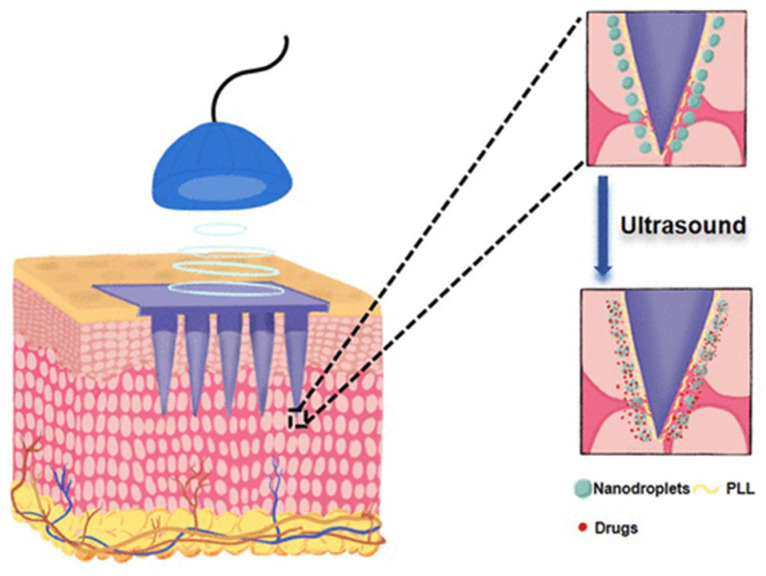
Schematic overview of US activation of nanobubbles. Schematic shows the penetration of the nanobubbles through the microneedles inserted transdermally and activated through US bursting. Reprinted (adapted) with permission from Ref. [95]. Copyright 2022, American Chemical Society.

Microbubbles can also be delivered into local areas of the body in addition to being delivered systemically. Local administration is often performed for special use cases of microbubbles and NBs. Corthesy et al. and Bioley et al. discussed that MBs could be administered nasally or orally as a way to provide mucosal vaccinations [93]. However, MBs can be unstable, and some improvements to their structures may need to be made before this method is widely adopted. NBs, on the other hand, have shown promise for transdermal administration [94,95]. Normally drugs that are given transdermally are limited by their ability to diffuse through interstitial fluid. However, NBs that are added into a microneedle patch and used in addition to the US cause better penetration and diffusion of drugs, as depicted in Figure 8 [95]. 

## 5. Medical Bubble Applications

The use of MNBs, especially with the US guidance, has continued to grow in its applications for diagnostic use and treatment of cancer, acute/chronic illnesses, and genetic disorders. Clinical and laboratory trials have shown very promising results, but further studies and research speculations are needed to conclude MNBs’ long-term effectiveness and safety profile. 

### 5.1. Cancer

Despite advances in preventative interventions, evidence-based screenings, and improved treatment regimens, cancer continues to be a leading cause of mortality worldwide, accounting for nearly one in six deaths [96]. Cancer therapies, such as tumor resection, chemotherapeutics, and radiation, are shown to be successful in inducing cancer remission but have numerous reported side effects and toxicities, even when being linked as modulators of secondary cancers. In efforts to mitigate these unintended effects, new classes of antineoplastics and advanced therapeutics are frequently being developed.

Scientists continue to close the knowledge gap in what is known about cancerous mechanisms, which has allowed clinicians to target cancer cells more selectively and personalize treatment regimens. One such innovation has been the use of MNBs with the US. MNBs can be infused with an antineoplastic agent, introduced to the body, and directed via ultrasonic waves to selectively target the cancerous area of interest. These gaseous spheres withstand different protective layers of the body and then undergo sonoporation, a process in which the MNBs decompose and de-load the therapeutic mixture when exposed to US waves. This is especially useful for chemotherapeutic agents because it decreases their impact on surrounding structures while optimizing their amount within malignant tissues.

One clinical case that demonstrated the unique potential of MBs was their use for pancreatic cancer. The combination of MBs and gemcitabine alongside the US showed a significant decrease in tumor growth, which is not typical for this particularly aggressive and resistant cancer [97]. MBs can also be coated with substances to aid in the targeting of specific tumor regions, allowing for further precision. It was shown that lipid MBs loaded with the chemotherapeutic drug paclitaxel (PTX), and coated with the breast tumor homing peptide, LyP-1, reached sufficient concentrations and adequate drug-loading efficacy [35]. In a similar fashion, PTX MBs that were designed to target vascular endothelial growth factor (VEGF) successfully induced apoptosis of the human breast cancer cell line (MCF-7) and suppressed their proliferation [98]. In both scenarios, the targeted PTX-loaded MBs were used in conjunction with the US for final drug delivery via induced rupture once they reached their targeted location. Similarly, MNBs can be loaded with antineoplastic agents and oxygen simultaneously, resulting in further anticancer efficacy [16,99,100]. Given that oxygen therapy can improve medication absorption, increase therapeutic ability, and better withstand hypoxia-induced tumor cell resistance, the co-delivery of chemotherapeutics and oxygen via US-guided MNBs is a synergistic method worthy of further inquiry. 

### 5.2. Osteoporosis

Osteoporosis is a condition in which the bones become weak and brittle due to an imbalance between bone resorption and new bone regeneration. While there are multiple etiologies that contribute to the development of osteoporosis, the risk increases with age and is more common in women as they lose protective estrogenic effects. Osteoclasts and osteoblasts are the two main cellular players that are targeted under study for the development of treatments to delay/reverse the osteoporotic process. Osteoclasts are responsible for aged bone resorption, and osteoblasts are cells that construct new bone. In osteoporosis, these cells are no longer coordinating in synchrony. Often, the symptoms of osteoporosis are silent until a pathologic fracture occurs due to the decreased bone density. Because osteoporosis can be a debilitating disease, advanced treatment strategies are currently being investigated, including the use of MNBs as selective drug/gene deliverers.

One study demonstrated how the US-directed nanodroplets, embedded with alendronate and encapsulated with osteoporosis-related silencing gene Cathepsin K small interfering RNA (CTSK-siRNA-ND-AL), successfully suppressed osteoclastogenesis [101]. Another study illustrated the protective effects of oxygen-infused nanobubbles (O2-NBs) on bone metabolism. Patients treated with long-term glucocorticoid therapies are at an increased risk of developing osteoporosis. It was shown that in mice models, the O2-NBs prevented glucocorticoid-induced bone loss by affecting cellular signaling [102]. The development of advanced strategies, such as selectively targeted NBs, to prevent osteoclastogenesis continues to be explored in efforts to mitigate the systemic effects of current oral/injectable therapies used to treat osteoporosis.

### 5.3. Management of Acute Medical Conditions

#### 5.3.1. Blood–Brain Barrier

The BBB is a tightly regulated, protective mechanism that has been a consistent obstacle for drug/gene therapeutics targeted for the CNS. The properties of MNBs, when introduced to the US, have been investigated as a possible method by which the BBB can be bypassed. The influence of the US on MNBs could potentially aid in the passage of therapeutic materials across the BBB to reach diseased areas of the CNS. When MNBs are met with ultrasonic waves, they oscillate and eventually decompose as the frequency of the waves increases. The rupture of MNBs has been shown to increase capillary and cell membrane permeability of nearby structures, allowing for medicinal cargo to slip by [35]. This is particularly useful because this method is minimally invasive and reversible.

Since NBs have a relatively smaller diameter than MBs, they can permit the passage of therapeutic substances while minimizing the impact on surrounding tissues. In one study, NBs demonstrated more acoustic control and consistency when compared with MBs [101]. The literature has also shown how NBs could potentially be used as novel thrombolytic agents. Patients with antiphospholipid are highly hypercoagulable because they produce antibodies that often target beta-2-glycoprotein (β2-GPI), a serum protein commonly bound to key players of thrombus formation [102]. A study revealed that targeted NBs coated with recombinant tissue plasminogen factor (rtPA) and recombinant antibody specific to β2-GPI preferentially bound to platelets, leukocytes, and endothelial cells within the thrombi [102]. When introduced to a rat model, rtPA-tNBs not only induced rapid thrombolysis but also prevented the formation of new thrombi. This type of method in humans will need to be further evaluated for safety and effectiveness, but the results of this study are encouraging for the future use of thrombolytic NBs. Because of their small diameters and ability to be selectively targeted, NBs as antithrombotic agent deliverers have the potential to reduce the bleeding risk, limit systemic effects, and improve the overall safety profile of thrombolytic agents.

#### 5.3.2. Lung-Targeted Delivery

While there is a plentiful amount of the literature supporting the utility of MNBs in drug delivery to tumor cells and across vascular regions, such as the BBB, their application for the treatment of pathologies of lung endothelium is quite scarce in comparison. The US cannot penetrate air-filled lung tissue due to scattering properties but can transmit through fluid-containing regions. Exceptional selectivity can be achieved through this mechanism since ultrasonic waves can locate the most damaged areas of the lung airways, which would be the locations of fluid leakage via the injury/infection [101]. This ability to discriminate between the air and the fluid/blood interfaces distinguishes the US-able MBs from other drug delivery methods. One study demonstrated that when introduced to an *Escherichia coli* (*E. coli*) murine pneumonia model, gentamicin-infused MBs had a 10-fold decline in bacterial colony-forming units compared with gentamicin in isolation [102]. Aminoglycosides typically do not dispense well within lung tissue. However, the MBs-gentamicin mixture with the US was able to maintain adequate concentrations. Gentamicin-infused MBs with a coating of liposomes and fluorescent markers were demonstrated to be a potentially more efficacious alternative for treating urinary tract infections (UTIs). The data of one study with a human urothelial organoid model revealed a 16-fold greater US-activated intracellular drug delivery via MBs than the control and 2-fold that of liposomes alone [103].

## 6. Bubbles in Clinical Trials

Clinical trials were evaluated using Clinicaltrials.gov. Clinical trials were looked at for MNBs. For NBs, there are five studies total after conducting a search of “Other terms: nanobubble” (Table 1). One was excluded as its status was marked as “Withdrawn” with 0 participants. For the MBs, however, there were 161 after conducting a search of “Other terms: microbubble”. After excluding “Observational”, “Patient Registries”, and “Expanded Access” studies and only allowing “Interventional Studies”, 134 studies were left. Next, studies that were listed as “Suspended”, “Terminated”, “Withdrawn”, and “Unknown status” were excluded, only allowing studies listed as “Recruiting”, “Not yet recruiting”, “Active, not recruiting”, “Completed”, and “Enrolling by invitation Studies”, resulting in 98 studies. Following that, studies with primary results before 2016 were excluded, leaving 79 studies. Lastly, studies were checked for relevance to MBs, with seven excluded, leaving a final of 72 studies. Of these 72 studies, 8 had completed results that were published (Table 2).

## 7. Bubble Challenges and Opportunities

### 7.1. Microbubble Design

MBs have a circulation time of approximately 10–15 min before being removed from the portal circulation by Kupffer cells [42]. The turnover time is dependent on numerous factors, including the size, gas core, and shell compositions [41,42,73]. The primary three-shell components are lipid-based soft shells, protein-based moderate shells, and polymeric hard shells.

Most of the commercially available US-contrast-enhancing MBs are lipid-shelled as they have favorable oscillation and contrast profiles; however, their heterogeneity, limited drug loading capacity, and low stability are barriers that are currently being explored [73]. Differing dispersions and solubilities have been experimented with, and recently, liposome dispersion has shown great promise in extending the half-life of lipid MBs with minimal cytotoxicity [71]. Additionally, multiple stabilizing agents are being tested, such as PEG (PEGylated- MBs), which have been shown to survive longer in blood and induce no immune response [104].

Polymeric hard shells have thicker shells which allow for more stability and higher amounts of molecules loaded but with the tradeoff of reduced contrast [65]. Due to this increased stability, chemical modifications are more easily designed on polymeric materials, with an example being the incorporation of sonosensitizers to further improve the therapeutic effect of the tumor cell oxidative damage [66]. Although polymeric MBs are traditionally poorly responsive to the US, recent studies have shown promise in designing next-generation polymeric vehicles that do not have this downside [105].

Protein MBs exhibit moderate properties in soft lipid shells and polymeric hard shells, with their major advantage pertaining to therapeutic and clinically relevant protein–protein interactions [41]. Human serum albumin (HSA) has been the most widely used protein shell; however, other proteins have recently been shown to be extremely effective. For example, the addition of the protein oleosin has been shown to have longer shelf times and to be more stable when exposed to the US, which shows great potential for delivering therapeutic gasses as well as prolonged US imaging [106].

### 7.2. Endothelial Barrier

MBs have shown great potential in interacting across the endothelial barrier, with one such way involving breaking the tight junctions between endothelial cells, a technique called sonopermeation. Helfield et al. and Beekers et al. have explored this process in vitro and have found that these gaps can stay open for tens of minutes, allowing a significant amount of drug delivery [107,108]. Although the level of calcium influx has been shown to predict the opening of these gaps, more research is needed regarding the role of chemical components on cells and tight junction poration as a whole [42].

Another major difficulty in targeted MBs in the endothelium has been the off-target effects that often happen in complex in vivo systems [109]. There have been a few different methods deployed to counter this issue, one of which is to select a receptor that is highly upregulated in the targeted tissue. Additionally, new target ligands are starting to be developed and tested for increased selectivity and decreased off-target effects; for example, Willman et al. used MBs conjugated to small peptide-targeting ligands to provide higher imaging signals than those provided by a large antibody molecule [110]. Additionally, Barreriro et al. proposed that combining multiple antibodies into the MB shell could enhance the specificity of the targeted MBs for endothelium expressing both targets, with further experimentation to come [111]. As further experimentation is conducted and it becomes easier to provide higher imaging signals, as well as target more specific sites, the off-target effects should decrease drastically.

Another challenge that needs to be further explored is increasing vascular permeability while balancing some of the associated disadvantages. Although increasing vascular permeability increases drug delivery, some of the disadvantages include hemorrhage, thrombosis, ruptures, and occlusion. It has been found that the probability of vascular rupture highly depends on the acoustic settings and, as expected, is less likely to happen in larger and thicker vessels [112]. Vasoconstriction and subsequent vascular shutdown can also lead to prothrombogenic factors, which can lead to platelet activation and thrombus formation [113].

### 7.3. Blood–Brain Barrier

The brain is one of the most difficult regions in the human body to access and uses specific trans-endothelial transporter molecules [114]. The presence of very specific tight junctions (zonulae occludentes), as well as a high number of P-glycoprotein efflux pumps continuously pumping drug molecules back into the vasculature, pose a challenge to overcome [1]. Additionally, due to the inhomogeneous and porous structure of the skull itself and the large impedance mismatch between the bone and soft tissues, Song et al. found a transmission coefficient of approximately 14% in humans and 50% in rats [115]. A few approaches have been developed to overcome these challenges, such as the time-reversal approach and patient-specific US beam profiling [42]. Both have shown promise in decreasing collateral damage but still have practical difficulties. 

Perhaps even more critical than in the endothelium, the issues of neuroinflammation and vascular damage need to be addressed when discussing the BBB. Kovacs et al. recently found that the opening of the BBB by the US and MB infusion resulted in immediate damage-associated molecular patterns that led to a sterile inflammation response within the parenchyma that lasted 24 h that was compatible with ischemia or mild TBI [116]. Additionally, several studies have shown astrocyte damage and/or astrocyte activation, which led to neurotoxicological damage [42]. However, the administration of dexamethasone has been shown to modulate the duration of BBB permeability enhancement and may reduce the risk of inflammation-induced tissue damage, increasing the safety profile of the US and MB delivery strategy [117]. Physiologically, the BBB has been designed to be hard to access, and safer methods need to be developed before US or MB methodologies can be effectively used.

### 7.4. Immunological Barrier

Although not a physical barrier, the immunological system has specific defenses that often need to be overcome to effectively treat with MBs and US techniques. This section will summarize specific strategies that have been utilized to complement or actively increase the effectiveness of the MB technology in regard to the body’s immune system. In the past decade, there have been numerous breakthroughs in immunotherapy, ranging from checkpoint inhibition therapy to APC processing, with subsequent antigen-specific T-cell proliferation [42]. Bioley et al. demonstrated that MB could successfully target APC both in vitro and in vivo and, thus, may serve as a potent Ag delivery system without a requirement for the US-based sonoporation with important future implications for both active immunity and tolerogenic mechanisms [118]. Furthermore, multiple studies have successfully delivered antigen peptides, pDNA, and mRNA leading to a suppressed tumor growth in mice xenograft models [119,120]. Specifically, Un et al. suggest that their gene transfection method could be suitable for DNA vaccination aimed at the prevention of metastatic and relapsed cancer [120]. Recently, the US has been explored in regard to T-cell transfusions, and there are three autologous anti-CD19 CAR T-cell products that are currently approved by the US Food and Drug Administration (FDA) as a third-line treatment and beyond for patients with relapsed or refractory LBCL: axicabtagene ciloleucel (axicel); tisagenlecleucel (tisacel); and lisocabtagene maraleucel (lisocel) [121]. With an ever-increasing number of treatments and drugs being developed, the immunological barrier needs to be further explored. 

## 8. Summary

In conclusion, there are limitless opportunities presented in the realm of MNBs as they have shown that they can be applied as “theranostic” agents. MBs have traditionally been used for the US techniques for imaging, and NBs can be used for similar purposes. Furthermore, NBs have also demonstrated their utility in cancer treatments and gene delivery systems, as well as in reducing toxic effects by localizing the administration of various treatments. Different dispensing modalities can also be utilized, including oral, topical, and intravenous administrations. MBs can also be effective in their own way through applications toward larger organs, such as the heart, kidney, and liver. Clinical trials continue to operate, with a small but growing number of trials in NBs and multiple in regard to MBs. Nevertheless, research has shown the multiple uses already available for MNBs through their use in imaging, drug delivery, and gene therapy. More specifically, MNBs have been and may very well become paramount to the treatment of chronic conditions, such as cancer and osteoporosis, as well as acute conditions, such as brain infections and UTIs. Ultimately, MNBs are on the horizon of important advances in radiology and in drug and gene delivery, especially in decreasing systemic toxicity effects that can occur through less localized methods.

## Figures and Tables

**Table 1:** Clinical Trial NB Studies. The table shows the studies, condition, intervention, enrollment, and primary outcomes and results for the NB studies.

Study	Identifier	Condition	Intervention	Enrollment	Primary Outcome and Results	Status
Contrast-enhanced Ultrasound (CEUS) For Intraoperative Spinal Cord Injury	NCT05530798	Spine Disease and degeneration; Spinal Stenosis and Injury; Spinal Cord Diseases, Injuries, and Compression	Device: Definity US Contrast	20	Use of contrast enhanced US to identify discrete areas of perfusion changes in the spinal cord of subjects undergoing spinal cord decompression	Not Yet Recruiting
Feasibility of the Vapor Nanobubble Technology for Malaria Diagnostics (MalariaSense)	NCT02672228	Malaria	Device: MalariaSense device	208	Hemozoin-generated vapor NB (H-VNB) amplitude thresholds among malaria infected and uninfected individuals	Completed (2015)
The Effect of RNS60 on ALS Biomarkers (RNS60)	NCT03456882	Amyotrophic Lateral Sclerosis (ALS)	Drug: RNS60	142	Pharmacodynamic biomarkers to measure the effect of RNS60 treatment on selected pharmacodynamic biomarkers in ALS patients concurrently treated with riluzole.	Completed (2020)
Micro/Nanobubbles (MNBs) for Treatment of Acute and Chronic Wounds	NCT05169814	Open Wound Wound Heal	Drug: Irrigation: MNB and Other: 0.9% Normal Saline Drug: Negative Pressure Wound Therapy with Instillation: MNB and Other: 0.9% Normal Saline	40	Wound total oxygen saturation level; Wound Size/Surface Area (cm^2^); Analysis of wound pH; Wound oxyhemoglobin and deoxyhemoglobin concentration levels; Analysis of wound concentration levels of multiple cytokines.	Recruiting

**Table 2:** Clinical Trial MB Studies with Completed Results. The table shows the studies, condition, intervention, enrollment, and primary outcomes and results for the MB interventional studies that met inclusion criteria with published results.

Study	Identifier	Condition	Intervention	Enrollment	Primary Outcome and Results
Sonazoid™ vs. SonoVue^®^ for Focal Liver Lesions during Pre- and Post-CEUS	NCT03335566	Liver Lesions	Drug: I.V bolus Sonazoid™ Drug: I.V bolus SonoVue^®^	424 (214 completed Sonazoid and 203 completed SonoVue)	% of Participants with accuracy improvement post- vs. pre-contrast US examination of liver lesions as malignant or benign against reference diagnosis (RD). Assessments were performed by 3 blinded readers. For Sonazoid: 19.07% (17–22.1%); For SonoVue: 18.50% (14.6–24.2%).
CEUS for Complex Kidney Lesion Diagnosis in Patients With Chronic Kidney Diseases (CKD)	NCT03196076	CKD and Cystic Kidney Disease	Drug: Perflutren	25 (5 healthy subjects and 20 with kidney lesions)	# of Participants With Change in Radiologist’s Lesion Evaluation to determine whether a lesion has progressed, regressed, or is stable. 5 Not Applicable (healthy). Of not healthy: ○ 11 stable (55%); ○ 1 Progression (5%); ○ 2 Regression (10%); ○ 1 Unable to assess (5%); ○ 5 Not Applicable (25%).
Sonothrombolysis in Patients With ST—segment Elevation Myocardial Infarction (STEMI)	NCT03092089	STEMI	Drug: Definity Device: Myocardial Contrast Echocardiography Procedure: Reperfusion therapy with PPCI	15	# of Participants With Spontaneous Reperfusion as assessed by the following: ○ a pre-PCI ECG complete ST-segment resolution (>50%) (immediately prior to angiogram): of 11 analyzed, 2 immediate reperfusion seen; ○ a pre-PCI TIMI 2–3 flow on diagnostic angiogram (immediately prior to angiogram): Of 15 analyzed, 7 immediate reperfusion seen by pre PCI TIMI 2–3 flow.
Detection of High Grade Prostate Cancer With Subharmonic US Imaging	NCT02967458	Prostatic Neoplasm	Drug: Perflutren Lipid Microsphere Intravenous Suspension	55	% of Subjects Whose Prostate Cancer Was Detected With Subharmonic Imaging via increased visualization of prostate vascularity: 43.6%; % of Biopsy Cores in which Prostate Cancer was detected using subharmonic imaging: 8.33%; % of Subjects With previously unidentified prostate Cancer using magnetic resonance imaging (MRI): 9%* *only 31 participants analyzed
Real-Time Myocardial Perfusion Echocardiography (RMPE) for Coronary Allograft Vasculopathy	NCT02880137	Cardiac Allograft Vasculopathy	Drug: Perflutren Lipid Microsphere Definity Procedure: RTMPE IV biologically—inert MBs	36	# of subjects with a perfusion defect identified using the following: Clinically indicated invasive coronary angiography (ICA): 5/36 (13.9%); perfusion defect identified using (RTMPE): 6/36 (16.7%).
CEUS of the Kidney (CEUS-CKD)	NCT02684435	CKD; Cystic Kidney Disease	Drug: Perflutren lipid microsphere	63	# of lesions with change in radiologist’s evaluation assessed for change to determine whether a lesion has progressed, regressed, or is stable: 24 lesions; Sensitivity and specificity of qualitative interpretations of CEUS in diagnosing kidney malignancy/suspicious lesion on non-contrast imaging compared to the truth standard: ○ Sensitivity: 75% positive scans; ○ Specificity: 71% negative scans.
Noninvasive Subharmonic Aided Pressure Estimation of Portal Hypertension (HTN)	NCT02489045	Liver Diseases Portal Hypertension	Drug: Subharmonic aided pressure estimation (SHAPE) measurement (Sonazoid US contrast agent)	178 (125 completed) (53 not completed)	Diagnostic accuracy of SHAPE liver pressure estimates for diagnosing portal HTN with catheter pressure as reference standard: 95% (of 113 analyzed); SHAPE liver pressure estimates correlation with catheter pressure: 0.63 correlation coefficient (of 113 analyzed); SHAPE results vs. blood work and imaging in portal HTN patients to determine if SHAPE measurements can monitor disease progression or treatment response (Mean (SD)): ○ Of 10/11 participant responders: −4.01 (3.61); ○ Of 1/11 participant non-responders: 2.33 (0).
CEUS Sentinel Lymph Node Imaging With Guided Biopsy in Breast Cancer Patients	NCT02321527	Breast Cancer	Drug: Perflutren Protein-Type A Microspheres Injectable Suspension Device: CEUS Procedure: Biopsy + Radioactive Seed Placement	21 (20 completed) (1 not completed)	# of Breast Cancer Participants With Sentinel Lymph Nodes (SLN) Identification Using the CEUS Technique: 20/21 participants (95.2%)

## Data Availability

No new data were created.
